# Quality and duration of responses with mogamulizumab in cutaneous T‐cell lymphomas: Insights into long‐lasting outcomes

**DOI:** 10.1111/bjh.70161

**Published:** 2025-09-14

**Authors:** Alessandro Broccoli, Lisa Argnani, Alessandro Pileri, Camilla Mazzoni, Benedetta Sordi, Nicola Pimpinelli, Paolo Fava, Pietro Quaglino, Elsa Pennese, Tommasina Perrone, Andrea Bernardelli, Carlo Visco, Miriam Teoli, Leonardo Flenghi, Adalberto Ibatici, Cesare Massone, Pier Luigi Zinzani

**Affiliations:** ^1^ IRCCS Azienda Ospedaliero‐Universitaria di Bologna, Istituto di Ematologia “Seràgnoli” Bologna Italy; ^2^ Dipartimento di Scienze Mediche e Chirurgiche Università di Bologna Bologna Italy; ^3^ Dermatology Unit IRCCS Azienda Ospedaliero‐Universitaria di Bologna Bologna Italy; ^4^ Division of Haematology, Department of Clinical and Experimental Medicine University of Florence Florence Italy; ^5^ Division of Dermatology, Department of Health Sciences University of Florence Florence Italy; ^6^ Dermatologic Clinic, Department of Medical Sciences University of Turin Medical School Torino Italy; ^7^ UOC Ematologia Clinica, Dipartimento Oncologico‐Ematologico, PO Santo Spirito Pescara Italy; ^8^ UOC Ematologia con Trapianto, AOU Policlinico di Bari Bari Italy; ^9^ Haematology and Bone Marrow Transplant Unit, Department of Medicine Azienda Ospedaliera Universitaria Integrata di Verona Verona Italy; ^10^ Porphyrias and Rare Diseases Unit San Gallicano Dermatogical Institute, IRCCS Rome Italy; ^11^ Azienda Ospedaliera di Perugia Perugia Italy; ^12^ Haematology and Transplant Unit IRCCS Ospedale Policlinico San Martino Genoa Italy; ^13^ Dermatology Unit Galliera Hospital Genoa Italy

**Keywords:** mogamulizumab, mycosis fungoides, Sézary syndrome

To the Editor,

Mycosis fungoides (MF) and Sézary syndrome (SS) are the most common cutaneous T‐cell lymphomas, both presenting by definition with skin involvement at different extent, from localised patches and plaques to nodular tumour lesions and diffuse erythroderma. Nodal or visceral infiltration may sometimes be present along with variable peripheral blood involvement by neoplastic lymphocytes that show a characteristic cerebriform nucleus, thus deserving the appellation of *monstrueuses*, as stated by Sézary and Bouvrain in one of their first reports.^1^ Both MF and SS are incurable: treatment is therefore considered palliative or, in other words, it should be directed to the management of symptoms and the control of disease burden.

Mogamulizumab, a first‐in‐class anti‐C‐C chemokine receptor 4 monoclonal antibody, has proven to be effective in the treatment of patients with MF and SS receiving at least one prior systemic treatment. It has confirmed its superiority over vorinostat—a histone deacetylase inhibitor—across any disease stage in the MAVORIC phase 3 randomised study, predominantly in advanced disease (stage IIB–IV), displayed by more than three quarters of patients enrolled in the trial.^2^ Objective responses with mogamulizumab were reported in 28% of treated cases by global assessment, with higher efficacy in SS and the deepest impact on the blood compartment. Patients showed a median progression‐free survival (PFS) of 7.7 months, in comparison to a median PFS of only 3.1 months observed in those who received vorinostat.^2^

Besides these favourable results, data on long‐term outcomes for those who achieve and maintain a durable response after mogamulizumab are very limited: only 11% of patients enrolled in MAVORIC achieved responses lasting more than 1 year,^2^ despite the more advanced the disease (especially in terms of circulating burden),^3^, ^4^ the higher the quality of the response.

With the purpose of investigating outcomes associated with durable responses in the real‐life setting, we have collected data on 21 patients treated at nine institutions in Italy with mogamulizumab according to the standard schedule who achieved a global objective disease remission, consisting of either a complete response (CR) or partial response (PR), lasting at least 4 months. These patients have been extrapolated from a total of 121 patients receiving mogamulizumab at the same institutions between 2015 and 2024, with the majority (more than 90%) treated since 2021 as soon as the drug was formally approved in Italy. We focused on patients with an objective response lasting at least 4 months in order to capture both the efficacy of the treatment (i.e. depth of response) and the meaningfulness of response duration in a single parameter, as performed by investigators in the earlier ALCANZA trial with brentuximab vedotin.^5^ Disease staging and response categorisation were performed according to internationally acknowledged recommendations.^6^ Demographics and patients' characteristics were summarised by descriptive statistics (Table 1) and survival functions were estimated by the Kaplan–Meier method.

**TABLE 1:** Patient characteristics and treatment outcomes.

Patients	21
Sex	
Male	13 (61.9%)
Female	8 (38.1%)
Age group at mogamulizumab start	
<70 years	8 (38.1%)
≥70 years	13 (61.9%)
≥75 years	9 (42.9%)
Median time from diagnosis to mogamulizumab	29.3 (3.6–185.3) months
Disease presentation	
MF	5 (23.8%)
SS	16 (76.2%)
Stage	
IIA	2 (9.5%)
IIB	2 (9.5%)
IIIB	3 (14.3%)
IVA_1_	14 (66.7%)
Skin involvement	
T_2_	4 (19.0%)
T_3_	2 (9.5%)
T_4_	15 (71.4%)
Blood involvement	
B_0_	3 (14.3%)
B_1_	4 (19.0%)
B_2_	14 (66.7%)
Circulating Sézary cells, mean value	
B_0_	160 cells/mm^3^
B_1_	350 cells/mm^3^
B_2_	3816 cells/mm^3^
Nodal involvement	
N_0_	8 (38.1%)
N_1–2_	5 (23.8%)
N_X_	8 (38.1%)
Visceral involvement	
M_0_	21 (100%)
M_1_	0
Best global response	
Complete	12/21 (57.1%)
Partial	9/21 (42.9%)
Skin response	
Complete	12/21 (57.1%)
Partial	8/21 (38.1%)
Stable disease	1/21 (4.8%)
Blood response	
Complete	16/18 (88.9%)
Partial	2/18 (11.1%)
Nodal response	
Complete	13/13 (100%)

Abbreviations: MF, mycosis fungoides; SS, Sézary syndrome.

Sixteen patients had SS and five MF; the predominant stage was IVA_1_ (66.7%), with B_2_ blood involvement in 66.7% of cases. Median age at mogamulizumab start was 73.6 years (range: 50.2–88.5) and 42.9% of patients were older than 75. Median time from MF or SS diagnosis to treatment initiation was 29.3 months (range: 3.6–185.3), after a median of 3 (range: 1–7) previous systemic therapies, including steroids in 71.4% of the cases.

Long responders to mogamulizumab accounted for 17.4% of all treated patients according to our definition. With a median number of 17 administrations, patients achieved a CR and a PR in 57.1% and 42.9% of the cases, respectively, and displayed a median time to response of 7.7 months. Nearly 81% of patients obtained a response within 2 years of treatment. According to compartments, CRs observed in the skin, blood and nodes represented 57.1%, 88.9% and 100% of the cases respectively. No patient had visceral (M_1_) dissemination before treatment.

At a median follow‐up of 35.3 months, 67.0% of patients were ongoing responders at 3 years since treatment start. Moreover, 67.9% were free of any subsequent treatment. Median overall survival (OS) was not reached, and the 5‐year projected OS was 70.8% (Figure 1).

**FIGURE 1:**
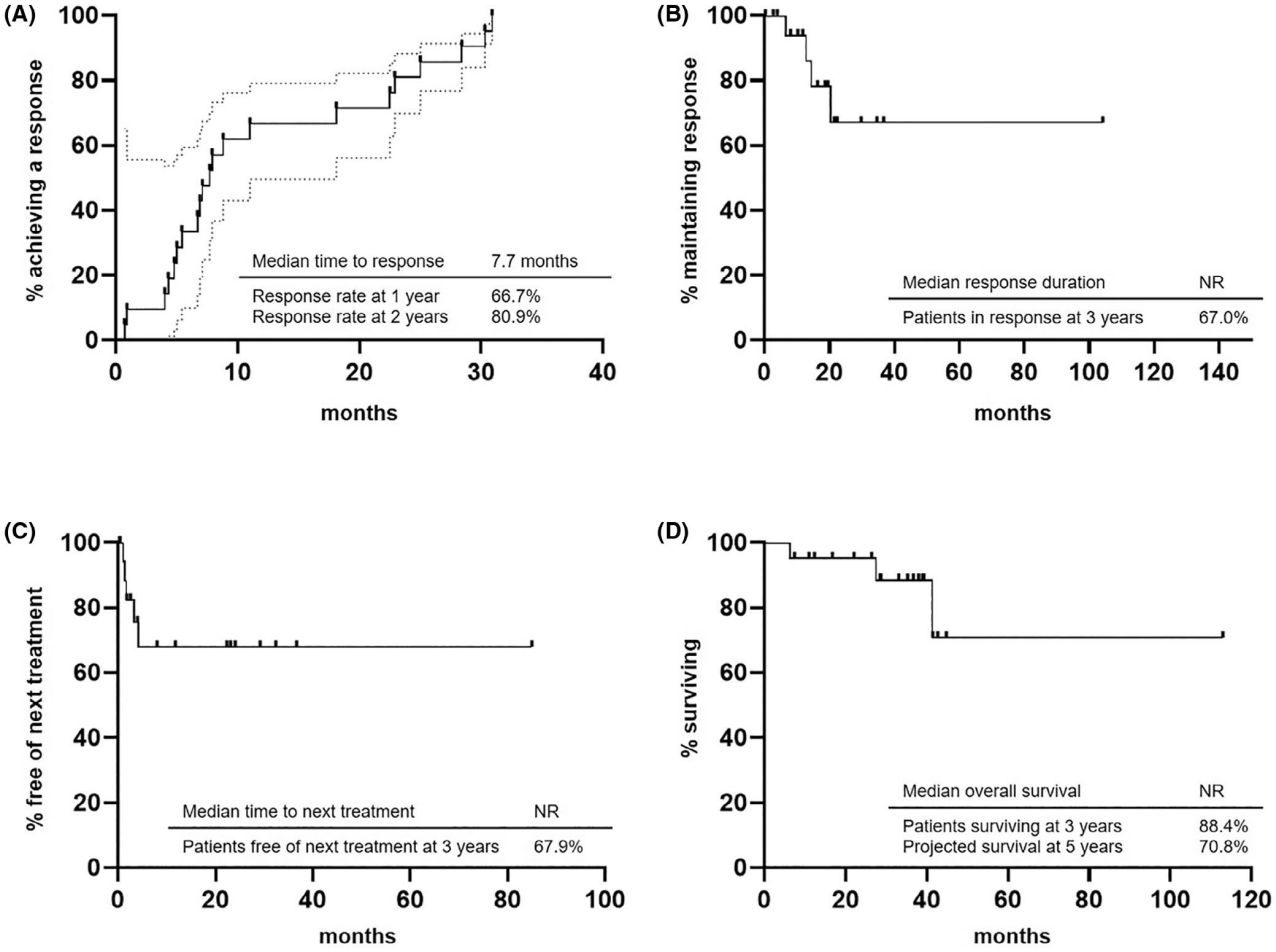
Time to response (A), duration of response (B), time to next treatment (C) and overall survival (D) for the entire cohort of patients.

Nine patients (42.9%) developed mogamulizumab‐associated rash (MAR), which represented the cause of treatment discontinuation in three cases. MAR developed at a median of 6.4 months (range: 2.4–26.1) since mogamulizumab start and had a median duration of 4.5 months (range: 0.5–15.3). We observed no correlation between MAR development and response duration.

In terms of haematological toxicity, one patient developed grade 1 drug‐correlated thrombocytopenia and one had grade 1 mogamulizumab‐unrelated pancytopenia. In both instances, cytopenias fully recovered within 10 days without interventions or sequelae. On the extrahaematological ground, there were 11 adverse events (AEs) in six patients, including grade 3 non‐cardiac thoracic pain and drug‐related pruritus without MAR, both causing treatment discontinuation; grade 2 thyroiditis, varicella zoster reactivation and pruritus; grade 1 photosensitivity, upper respiratory tract infection, COVID‐19, alanine aminotransferase increase and constipation (the latter in two cases).

Eleven patients discontinued mogamulizumab: apart from MAR and AEs, three patients went off treatment because of disease progression or death and two due to other causes (patient's wish and progression of synchronous pancreatic adenocarcinoma). One patient stopped mogamulizumab as he received allogeneic stem cell transplantation (allo‐SCT). Five patients (23.8%) received further treatment after mogamulizumab, including bexarotene (two cases), brentuximab vedotin, interferon and the aforementioned allo‐SCT (one case each).

Freedom from progression and probability of survival in MF and SS patients is strictly dependent on disease stage at presentation and correlates to skin and circulating disease burden.^7^, ^8^ Stage dictates treatment approach: skin‐directed treatments are suitable for localised disease, while systemic therapies are required in case of advanced disease or as soon as topical approaches demonstrate failure.^9^ PFS rates, however, turn out to be dismal in multitreated patients with advanced‐stage disease, as demonstrated by the control arms of the two most powerful phase 3 randomised studies, ALCANZA^5^ and MAVORIC.^2^ Briefly, ALCANZA was designed to test brentuximab vedotin against a physician's choice of methotrexate and bexarotene (conceived as the best available treatment options) after at least one previous line of therapy in patients with MF, excluding the SS presentation. In this study, median PFS with best available therapy was 3.5 months, significantly overcome by brentuximab vedotin, which yielded a median PFS of about 17 months.^5^ Likewise, PFS in vorinostat‐treated patients, according to MAVORIC, was 3.1 months, with a proportion of responding patients of only 5%.^2^ Given that MF and SS are both incurable, treatments should be aimed at pursuing the best response quality for as long as possible, with minimal impact on quality of life, if no impairment at all.^10^ Our data indicate that objective responses obtained with mogamulizumab can be durable, with no significant treatment‐emergent AEs that jeopardise patients' compliance. Importantly, patients who achieve an adequate disease control may remain in response longer than 3 years, without switching to another agent in more than 65% of the cases and with no detrimental impact on survival rates, even if highly pretreated and presenting with advanced‐stage disease. Higher efficacy of mogamulizumab in SS patients, especially in cases with high circulating tumour burden, is confirmed in our series, as previously described^3^ and in line with real‐life data published so far.^11^, ^12^, ^13^ MAR has shown a positive influence on response quality and duration in MAVORIC as well as in everyday clinical experiences.^14^, ^15^ Notably, the incidence of MAR in our series of fully responding patients reaches 42.9%, although from the present study we cannot establish any correlation between MAR and the achievement of longer responses.

In conclusion, responses obtained with mogamulizumab in highly pretreated patients with MF and SS can be long‐lasting, with a significant impact on disease burden in any of the involved compartments, most of all on skin and peripheral blood. The achievement of a durable response is a clinically meaningful outcome in patients affected by incurable diseases that severely impair their quality of life.

## AUTHOR CONTRIBUTIONS

Al.Br. and P.L.Z. conceived the study; Al.Br., L.A. and P.L.Z. wrote the manuscript; A.P., Ca.Ma., B.S., N.P., P.F., P.Q., E.P., T.P., An.Be., C.V., M.T., L.F., A.I. and Ce.Ma. provided study data; L.A. analysed the data; all authors read and approved the final version of the manuscript after revising it critically. All authors have access to the final database.

## FUNDING INFORMATION

The work reported in this publication was funded by the Italian Ministry of Health, RC‐2025‐2797392 project.

## CONFLICT OF INTEREST STATEMENT

Authors declare they have no conflict of interest pertaining to this work to disclose.

## ETHICS APPROVAL STATEMENT

The study had an official approval by the local Ethical Committee (approval ID 1043/2021/Oss/AOUBo).

## PATIENT CONSENT STATEMENT

Written informed consent was always obtained prior to collecting and analysing patients' data.

## Data Availability

The data that support the findings of this study are available from the corresponding author upon reasonable request.
